# Oleic Acid Promotes the Biosynthesis of 10-Hydroxy-2-decenoic Acid via Species-Selective Remodeling of TAGs in *Apis mellifera ligustica*

**DOI:** 10.3390/ijms241713361

**Published:** 2023-08-29

**Authors:** Xiyi Hu, Ying Wang, Xuepeng Chi, Hongfang Wang, Zhenguo Liu, Lanting Ma, Baohua Xu

**Affiliations:** 1 College of Animal Science and Technology, Shandong Agricultural University, Tai’an 271018, China; huxiyi@lyu.edu.cn (X.H.); wangying@sdau.edu.cn (Y.W.); xixuepeng@126.com (X.C.); wanghongfang22@163.com (H.W.); lzhenguo_001@163.com (Z.L.); malanting139@163.com (L.M.); 2 College of Agriculture and Forestry Science, Linyi University, Linyi 276000, China

**Keywords:** *Apis mellifera ligustica*, oleic acid, mandibular gland, 10-hydroxy-2-decenoic acid, lipidomics

## Abstract

This study aimed to assess the impact of oleic acid (OA) supplementation on the biosynthesis of 10-hydroxy-2-decenoic acid (10-HDA) in *Apis mellifera ligustica*. In experiment 1, varying concentrations of OA (2%, 4%, 6% and 8%) were added to an artificial diet for newly emerged bees reared in cages. Analysis of 10-HDA content and gene expression in the mandibular gland (MG) revealed that the 8% OA treatment had the greatest impact on promoting the synthesis of 10-HDA. Subsequent investigations utilized RNA-seq and lipidomics to characterize the molecular signature in the MG after feeding the 8% OA diet. Phosphatidylcholine (PC) and triacylglycerol (TAG) were found to be the predominant lipids in the MG of worker bees. A total of 154 TAGs were identified, with TAG (18:1-18:1-18:1) exhibiting the highest abundance, which increased by 1.5 times. The major TAG species contained palmitic acid (16:0) and oleic acid (18:1) in their structure, which was associated with fatty acid composition of diet. The increase in abundance of main TAGs may be attributed to the upregulation of glycerol-3-phosphate acyltransferase (Gpat) and glycerol kinase (GK) gene expression at the transcriptional level. The upregulation of differentially expressed genes (DEGs) related to carbohydrate metabolism may contribute to meeting the heightened metabolic demands of the MGs in worker bees. Royal jelly (RJ) samples from bee colonies fed with the 8% OA diet exhibited higher 10-HDA level than RJ collected from bee colonies fed with the artificial diet. These results indicate that 8% OA addition in the diet enhanced biosynthesis of 10-HDA in the mandibular gland, which was accompanied by significant and highly species-selective remodeling of TAGs.

## 1. Introduction

Lipids have key roles in insect physiological functions, including metabolism, reproduction, growth, and development. Further, lipids serve as structural components of cell membranes, as well as being used as an energy source, stored as energy reserves, and being involved in and regulating many physiological processes, including hormone and pheromone biosynthesis [1]. In honey bees, pollen is the nutritional source of lipids, and it also provides proteins, vitamins, and minerals [2,3,4]. Pollens differ in their nutritional content [3], including lipids [5] and fatty acid content and composition, which varies among different types of plants [3]. Bee bread is composed of 3–8% fats [6,7,8,9], whereas pollen lipids can be to up to 20% of the dry weight [3]. Pollen fatty acids help to maintain bee colony health and hygiene [5,10], and the three most common fatty acids in pollen are palmitic, linoleic (omega-6), and alpha-linolenic (omega-3) acids, which together comprise an average of 60–80% of all fatty acids in pollens [5,11,12]. These three fatty acids, in addition to oleic and stearic acids, are also the main fatty acids in honey bee bodies [11,12].

Pollen from various floral sources can affect bee health and the quality of their products since the nutritional content of pollen varies depending on plant type [13]. The mandibular and hypopharyngeal glands, which are located in the bees’ heads, are responsible for producing royal jelly (RJ). On a wet weight basis, RJ is composed of 60–70% moisture [14,15], 9–18% proteins [14], 3–8% lipids [14,15], 6–18% hydrocarbons [16,17], and 0.8–3.0% minerals [15]. According to a certain study, the lipids in RJ are mainly uncommon free fatty acids, which are up to 90% (i.e., hydroxyl and dicarboxylic fatty acids) [18]. Importantly, the fats in RJ are characterized by two large portion compounds: 10-hydroxy-2-decenoic acid (10-HDA) and 10-hydroxy-decanoic acid (10-HDAA), where 10-HDA is the primary fatty acid present in RJ [19], constituting up to 73% of the fatty acid profile (and up to 2–4% of RJ total wet weight). The most significant criteria in evaluating RJ for commercial trade is 10-HDA content, which varies with RJ origin and has been recommended as a metric indicating freshness. Among the criteria for RJ quality analysis, 10-HDA content varies with RJ origin and has been proposed as a parameter indicating freshness [19,20].

The lipid component of RJ is synthesized in the mandibular gland (MG) of nurse bees [21]. It primarily consists of organic fatty acids, with 10-HDA being the predominant one [22]. The MG of honey bee is an important exocrine gland that develops in all three castes (drone, queen, and worker) but serves distinct functions in each [23,24]. In queens, the MGs are responsible for producing decenoic acids hydroxylated at the ω-1-position, such as 9-oxo-2-decenoic acid (9-ODA) and 9-hydroxydec-2-enoic acid (9-HDA) [25]. These compounds play a crucial role as pheromones within honey bee colonies [26]. On the other hand, worker MGs primarily produce ω-hydroxylated decenoic acids, including 10-HDA and 10-HDAA [25,27], which are essential nutrition compounds for honey bee larvae [28]. For honey bees, oleic acid (OA) is an important fatty acid and is the prevailing fatty acid found in pollen from various species, particularly in the pollen of the fir tree species of the genus *Pseudotsuga* [5]. Fatty acids, including OA and palmitic acid, constitute the major portion of body fats in adult worker bees (60%), larvae (40%), and queen pupae (58%) [29]. Unsaturated OA is also dominant in lipid classes, while palmitoleic acid is present in substantial quantities across all classes of neutral lipids, such as triglycerides, sterols, sterol esters, and free fatty acids [5].

The purpose of this study was to investigate the effect of OA on the 10-HDA content of RJ. We applied lipidomics to unravel the changes in lipid metabolism in the MG after feeding a diet supplemented with OA. Lipidomics analysis enabled simultaneous analysis of changes in lipid profiles. Further, we performed transcriptome analysis by RNA sequencing to determine the molecular regulation of lipid metabolism genes during OA treatment.

## 2. Results

### 2.1. Determination of Average Feed Intake (AFI) in Nine-Day-Old Worker Bees Reared in Cages

There was no difference in AFI between groups during the trial period (Table 1). On days 0–2, the 8% OA group consumed less feed than the control group. On days 2–4, the 2% OA group consumed less feed than the control group, and on days 6–8, the 6% OA group consumed more feed than the control group. In general, all bees consumed more feed on days 2–4 across the whole period. On days 0–2, bee feed intake decreased with increased OA concentration, while on days 6–8, feed intake showed an upward trend accompanying increased OA concentration.

### 2.2. Determination of 10-HDA Content in Head and Gene Expression in Mandibular Gland of Nine-Day-Old Worker Bees Reared in Cages

**Figure 1:**
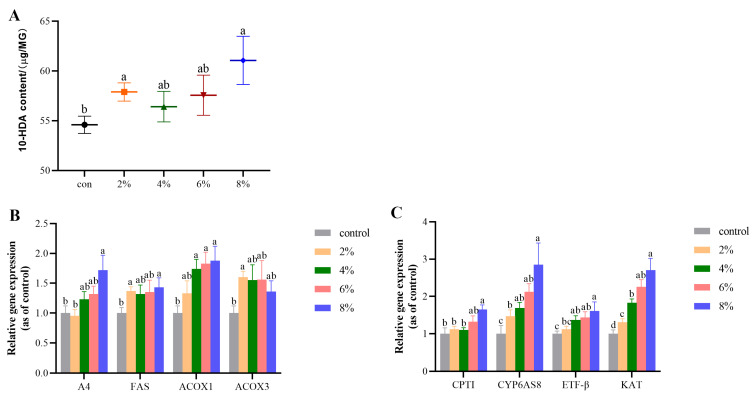
Effect of OA concentration (2%, 4%, 6%, and 8%) on 10-HDA synthesis. (**A**) 10-HDA content in head; (**B**) relative mRNA expression levels of *A4*, *FAS*, *ACOX1* and *ACOX3*; (**C**) relative mRNA expression levels of *CPT1*, *CYP6AS8*, *ETF-β*, and *KAT*. Values are expressed as means ± SEM (*n* = 8). Different letters (a, b, c, d) indicate significant differences (*p* < 0.05).

The results of analysis of 10-HDA content in head and gene expression in MG are presented in Figure 1. OA treatment (2% and 8%) increased the 10-HDA content, and the 8% OA group had the highest 10-HDA content (Figure 1A). Compared with the control group, 8% OA treatment increased expression of the genes, apolipophorin-III (*A4*), fatty acid synthase (*FAS*), acyl coa oxidase 1 (*ACOX1*), carnitine palmityltransferase-1 (*CPT1*), *CYP6AS8*, electron transfer flavoprotein β (*ETF-β*), and 3-ketoacyl-CoA thioase (*KAT*) (Figure 1B,C). Further, bees in the 2% OA group had higher mRNA level of *FAS*, acyl coa oxidase 3 (*ACOX3*), and *KAT*, compared with controls (Figure 1B,C). In addition, 4% and 6% OA supplementation increased expression of the *ACOX1*, *CYP6AS8*, and *KAT* genes relative to the control group (Figure 1B,C).

### 2.3. Non-Targeted Lipidomics Analysis of the MG in Nine-Day-Old Worker Bees Reared in Cages

**Figure 2:**
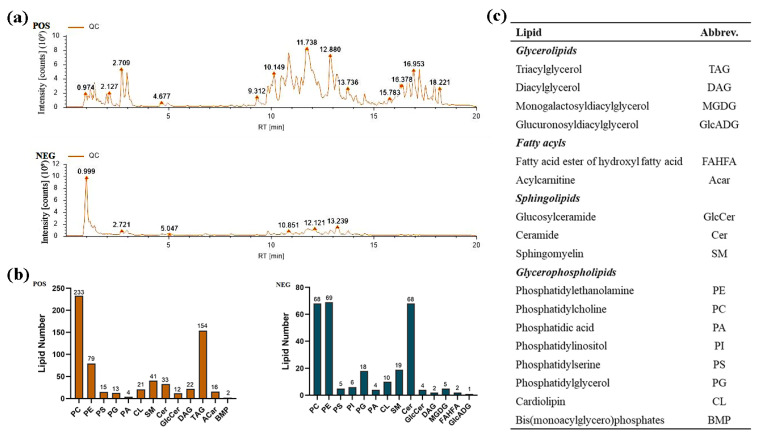
Lipidomics analysis of mandibular gland samples. (**a**) Total ion chromatogram (TIC) showing lipid profiles in mandibular gland acquired using UPLC-Q-Exactive Orbitrap MS (ESI+ and ESI−). (**b**) Number of lipids detected using a non-targeted analysis method in positive and negative ion modes. (**c**) Quantified lipid classes and their abbreviations used throughout the paper.

**Figure 3:**
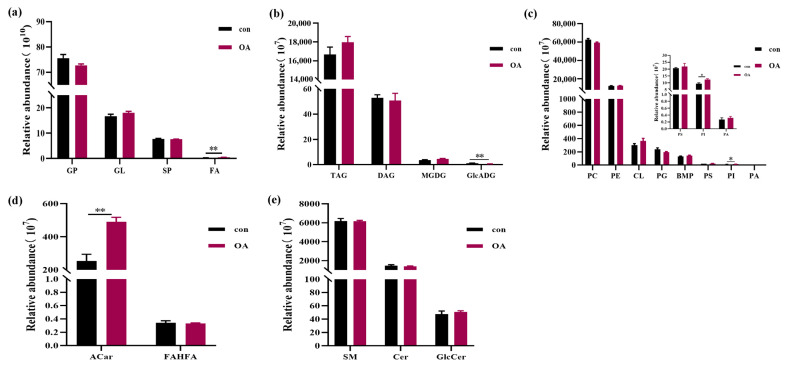
Total abundance of four categories (glycerolipids, glycerophospholipids, fatty acyls, and sphingolipids) in the mandibular gland (**a**). Abundance of the lipid classes of glycerolipids (**b**), glycerophospholipids (**c**), fatty acyls (**d**), and sphingolipids (**e**). Values are expressed as means ± SEM (*n* = 4). * *p* < 0.05 and ** *p* < 0.01.

To assess which lipidomic changes were associated with increased 10-HDA levels in the MG, we prepared lipid extracts from MGs of worker bees fed with a control artificial diet and diet supplemented with 8% OA, and performed non-targeted lipidomics analysis. In this study, a total of eight samples were analyzed using lipidomics analysis, including four replicates. Each sample consisted of 180 workers, with a total of 1440 individuals included in the experiment. All samples were analyzed in both positive- and negative-ion electrospray modes to ensure the identification of a comprehensive set of lipids [30]. A total of 1424 mass spectrometry features were detected from the raw data, including 983 positive peaks (representing specific lipid structures) and 441 negative peaks. A representative total ion chromatogram of the lipids from the MG samples is shown in Figure 2a. Across all samples, a total of 1424 unique lipid species were confidently identified, spanning four lipid categories and 17 lipid subclasses. The most commonly identified lipid species in the MG samples were of the phosphatidylcholine (PC) subclass, with 301 identifications, followed by the triacylglycerol (TAG) and phosphatidylethanolamine (PE) subclasses, with 154 and 148 identifications, respectively (Figure 2b). Quantification of the four lipid categories shown in Figure 3a revealed significant OA-induced changes in the abundance of fatty acyls (FA). The overall abundance of PCs and TAGs, which were the most and second-most abundant lipid classes in MGs, respectively, were not affected by 8% OA treatment (Figure 3b,c). In contrast, increased acylcarnitine (ACar) and phosphatidylinositol (PI) abundance and decreased glucuronosyldiacylglycerol (GlcADG) abundance were detected in response to OA supplementation (Figure 3b–d).

**Figure 4:**
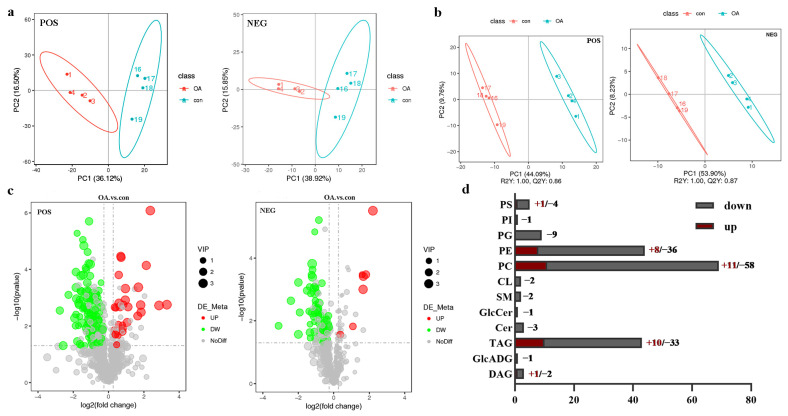
(**a**) PCA plots comparing lipid composition in the two experimental groups. (**b**) PLS-DA plots comparing lipid composition in the two experimental groups. (**c**) Volcano plots of different lipid species detected in the two experimental groups. (**d**) Lipid groups differentially expressed in bee mandibular glands from the two experimental groups. Total levels of lipids in each tissue type are shown next to the bars.

Next, we applied principal component analysis (PCA) and, partial least squares discrimination analysis (PLS-DA) to highlight the major compositional differences between the two groups (Figure 4a,b) and examined the altered lipids in detail. Figure 4a,b showed that the OA group was completely separated from the control group, indicating that OA induced altered lipid metabolism in MGs. We noted striking differences in MG lipid composition. Relative quantification of the identified lipids revealed that 219 species varied significantly (*t*-test; *p* < 0.05; Supplementary Table S4). The majority of altered lipids belonged to three lipid classes, comprising PC, PE, and TAG lipid species (Figure 4d). As shown by the cluster heatmap (Figure S2), in negative ion mode, 66 lipids differed; 7 lipids showed an increasing trend in the OA group, while 59 lipids showed a decreasing trend (Figure S2a). Further, in positive ion mode, 153 different lipids were detected, with 32 increased and 121 decreased in the OA group (Figure S2b). These differences in lipids between the two group are clearly illustrated by the heatmap shown in Figure S2.

**Figure 5:**
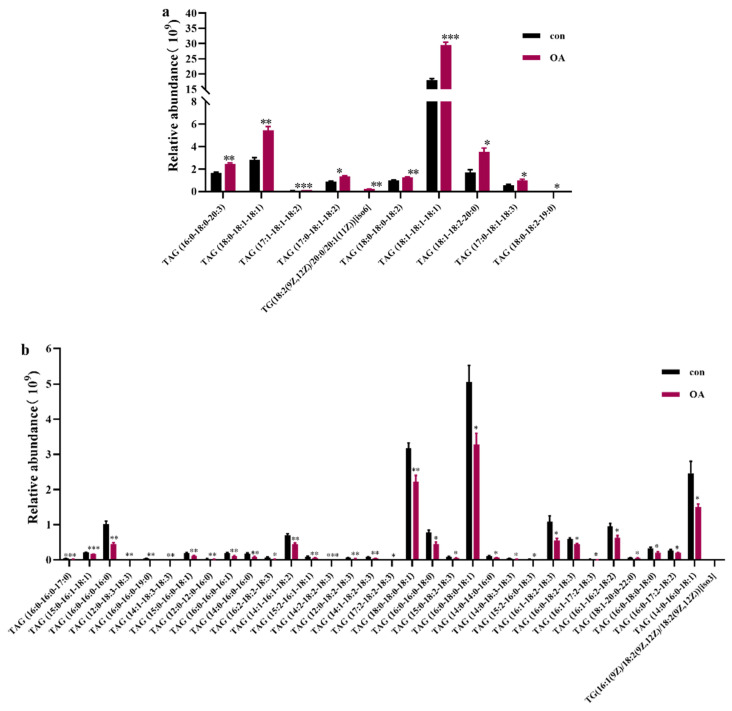
Relative abundance of (**a**) increased and (**b**) decreased TAG. Values are expressed as means ± SEM (*n* = 4). * *p* < 0.05, ** *p* < 0.01, and *** *p* < 0.001.

TAGs are the predominant lipid form in insects, functioning as energy storage molecules and as precursors for other molecules, such as semiochemicals [31]. A total of 154 TAGs were identified; TAG (18:1-18:1-18:1), TAG (16:1 (9Z)/18:1 (9Z)/18:1 (9Z)) [iso3], TAG (16:0-18:1-18:1), TAG (16:1 (9Z)/16:1 (9Z)/18:0) [iso3], TAG (16:0-18:1-18:3), and TAG (18:1-18:2-18:2) were the predominant TAG species in the MG, accounting for 51.34% and 55.27% in the control and 8% OA groups, respectively (Table 2). These major TAG species contained 16:0 (palmitic acid) and 18:1 (OA) in their structures. OA (0.0367 g/100 g diet) and palmitic acid (0.0214 g/100 g diet) were the first- and second-most abundant fatty acids in the artificial diet, respectively (Supplementary Table S2). These results indicate that the acyl chains of these major TAGs are related to dietary fatty acid composition. The concentrations of 10 kinds of TAGs increased, while those of 33 kinds of TAGs decreased. Details are shown in Figure 5. The abundance of TAG (18:1-18:1-18:1), which was the most abundant TAG species, increased by 1.64-fold.

### 2.4. Transcriptome Analysis of MG from Nine-Day-Old Worker Bees Reared in Cages

#### 2.4.1. Overview of Transcriptome Sequencing Results

A total of six *Apis mellifera ligustica* MG cDNA libraries were constructed for Illumina sequencing, including from the control (*n* = 3, 60 paired MG per replicate) and OA-supplemented (*n* = 3, 60 paired MG per replicate) groups. Sequencing data are summarized in Supplementary Table S5, and an overview of transcriptome sequencing results is presented in Table 3. Obtained Q20 (%) and Q30 (%) values were all >92% in both the control and OA-supplemented groups. On average, 6.97 and 6.36 GB of clean data were generated from the control and OA groups, respectively. Of the clean data, 79.95–94.99% and 93.54–95.85% from the control and OA groups, respectively, mapped to the *A. mellifera* genome. These RNA-seq results indicated that the obtained data had high quality and were suitable for subsequent bioinformatic analysis. Based on the screening threshold, comparison of the two groups yielded 1121 up-regulated and 1112 down-regulated genes for OA vs. control (Figure S3a and Table S6). The expression profiles of differentially expressed genes (DEGs) in the two groups were analyzed, and the results of hierarchical cluster analysis are shown in Figure S3b. To verify the expression levels of the DEGs identified by RNA-seq, nine DEGs were randomly chosen for quantitative real-time PCR (qRT-PCR) analysis using the same RNA samples. The patterns of differential expression are consistent with the results from transcriptome data (Figure S1).

#### 2.4.2. Gene Function Enrichment Analysis of DEGs

**Figure 6:**
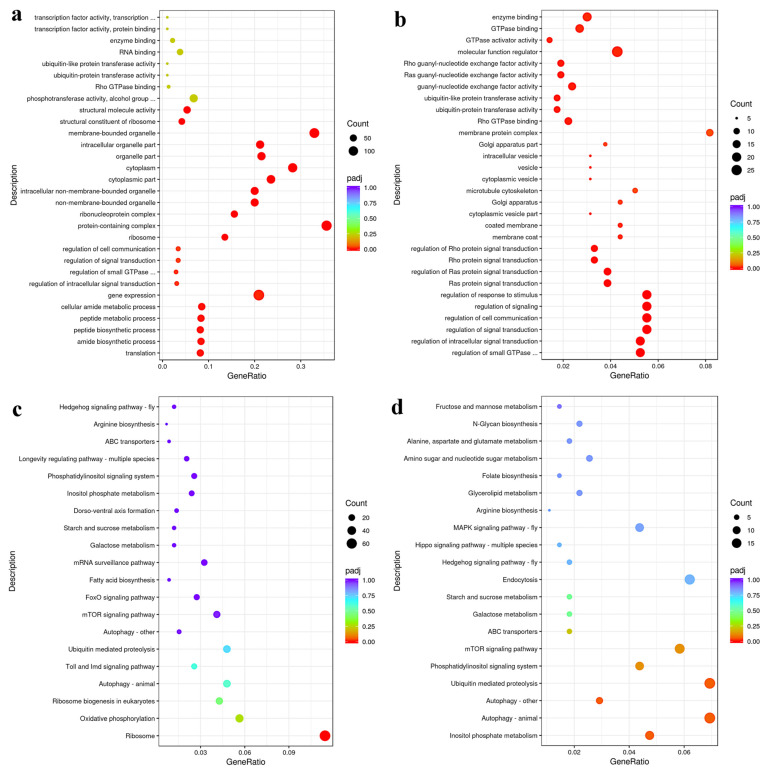
Top 30 GO biological processes, cellular components, and molecular functions enriched for differentially expressed genes (DEGs) (**a**) and differentially upregulated genes (**b**) for comparisons of OA vs. control groups. Top 20 KEGG pathways enriched for differentially expressed genes (DEGs) (**c**) and differentially upregulated genes (**d**) for comparisons of OA vs. control groups. Image description: the rectangle at right represents the saliency, and the colors from red to purple represent the decrease in saliency. Larger bubbles indicate higher numbers of DEGs.

To evaluate transcriptional changes in MG from the control and OA groups, Gene Ontology (GO) and Kyoto Encyclopedia of Genes and Genomes (KEGG) enrichment analysis were performed, based on the identified DEGs; the results are depicted in Supplementary Table S7. GO enrichment analysis identified 358, 80, and 227 GO terms enriched in “biological process” (BP), “cellular component” (CC), and “molecular function” (MF) domains, respectively; the top 30 GO terms in BP, CC, and MF are shown in Figure 6a. Of the top 10 GO terms in BP, GO annotation results are mainly enriched in “signal transduction”, “metabolic process”, and “biosynthetic process”. For upregulated DEGs, GO annotation results are clearly enriched in some signal transduction BPs as well as some terms related to vesicles (Figure 6b).

KEGG analysis identified 102 terms enriched for a total of 2233 DEGs. The top 20 enriched KEGG pathways are shown in Figure 6c, which suggests that “ribosome”-related functions dominated the most enriched pathways, while “fatty acid biosynthesis”, related to genes involved in lipid metabolism, was also an enriched pathway. Among upregulated DEGs, KEGG analysis showed that “inositol phosphate metabolism” and “autophagy” were the most enriched (Figure 6d). Additionally, in comparisons of the two groups, pathways involved in lipid metabolism (“glycerolipid metabolism”) as well as carbohydrate metabolism and “endocytosis” were specifically enriched.

#### 2.4.3. OA Drastically Changes the Expression of Genes Related to Glycerolipid Metabolism

**Figure 7:**
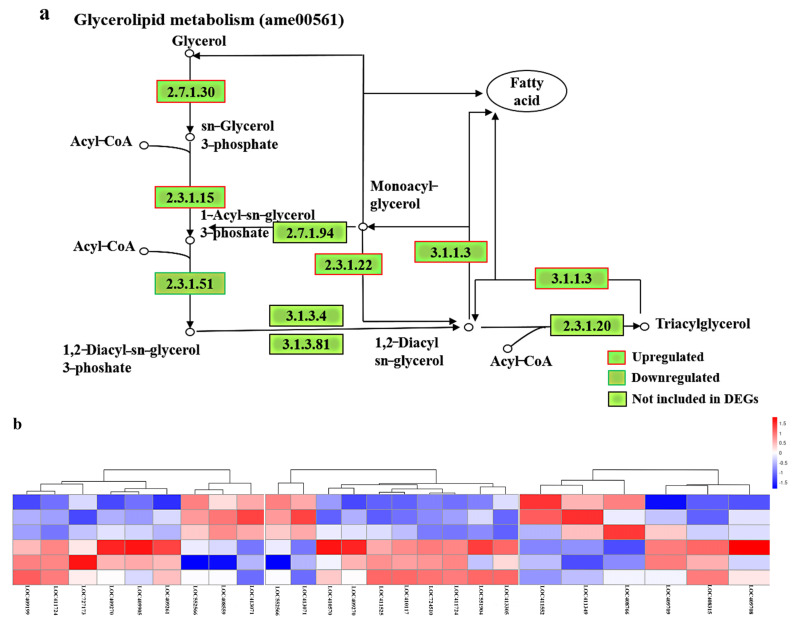
(**a**) KEGG pathway analysis of glycerolipid metabolism (ame00561), with selected key steps. (**b**) Hierarchical cluster heatmap of differentially expressed genes for three KEGG pathways. Glycerol kinase [2.7.1.30] (*GK*); glycerol-3-phosphate acyltransferase [2.3.1.15] (*GPAT*); lysophosphatidate acyltransferase [2.3.1.51] (*AGPAT*); triacylglycerol lipase [EC: 3.1.1.3] (*PL*); 2-acylglycerol O-acyltransferase 1 [2.3.1.22] (*MOGAT1*).

There was also evidence of alterations in the expression of genes involved in the lipid-associated pathway in the OA group. After feeding a diet supplemented with 8% OA, major TAG content was significantly increased relative to controls. Consistent with this, the expression levels of several genes involved in TAG synthesis were upregulated (Figure 7a), including *LOC411724* and *LOC 409270*, encoding glycerol-3-phosphate acyltransferase 1 (*GPAT1*) and glycerol-3-phosphate acyltransferase 4 (*GPAT4*), which were involved in the glycerolipid metabolism pathway and expressed at 1.24- and 1.29-fold higher levels in the OA group than in controls, respectively (Supplementary Table S6). *LOC 409261* and *LOC 409199*, encoding glycerol kinase (*GK*) in the glycerolipid metabolism pathway, were expressed at 1.42- and 1.27-fold higher levels in the OA group than in the control group, respectively (Supplementary Table S6).

Expression of *LOC727173*, encoding triacylglycerol lipase, was increased two-fold in the OA group. Together, these results indicate that OA treatment induced extensive changes in the glycerolipid metabolism transcriptome. These changes may contribute to higher secretory activity of the MG. Hierarchical cluster analysis of DEGs in three lipid-metabolism related KEGG pathways demonstrated that OA supplementation affected glycerolipid, glycerophospholipid, and sphingolipid metabolism, corresponding with dynamic changes in these lipids (Figure 7b).

### 2.5. Ultrastructural Analysis of MG in Nine-Day-Old Worker Bees Reared in Cages

**Figure 8:**
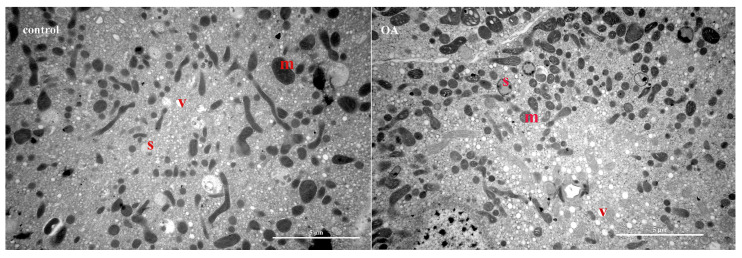
TEM images of mandibular glands in two comparison groups: s, secretory granules; m, mitochondria; v, vacuoles.

Transmission electron microscopy (TEM) demonstrated that secretory vacuoles of different sizes were predominant in the cytoplasm of MG cells, as well as large secretory granules with homogeneous content (Figure 8). In addition, numerous polymorphous mitochondria were also prominent components in the cytoplasm (Figure 8). Further, in control group workers, gland cells had fewer secretory vesicles in the cytoplasm (Figure 8). In contrast, in OA group worker bees, transparent vesicles in the cytoplasm were denser than those in controls, implying more vigorous secretory activity (Figure 8).

### 2.6. Feed Consumption and Colony Development

**Figure 9:**
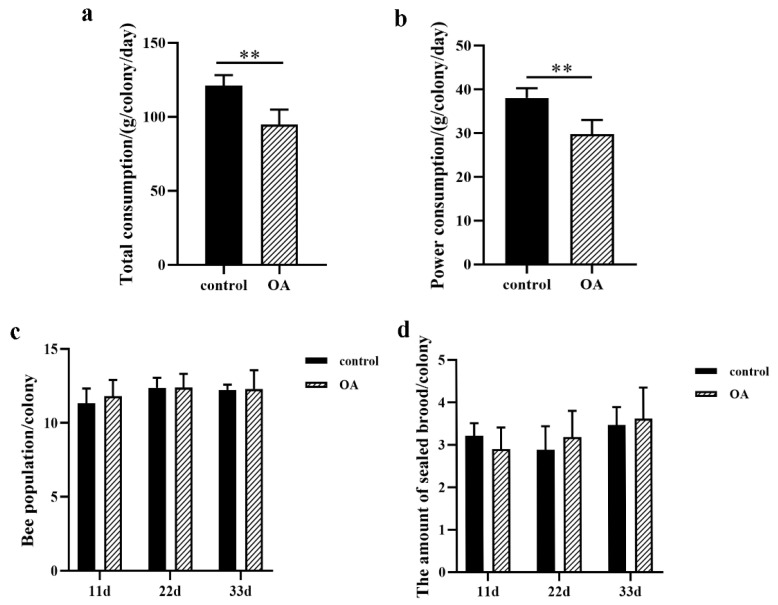
Feed intake of bee colonies receiving total mixture (**a**) and powder (**b**). Bee populations of colonies in the two experimental groups (**c**). Sealed brood development in colonies of the two experimental groups (**d**). Values are expressed as means ± SEM (*n* = 5); ** *p* < 0.01.

Dietary supplementation with OA resulted in a decreased feed consumption level relative to controls (Figure 9a,b). In addition, the 8% OA diet had no effect on bee development or sealed brood population (Figure 9c,d). Bee populations were approximately 12 frames per colony, with sealed brood populations of approximately three frames per colony.

### 2.7. RJ Production, 10-HDA Concentration in RJ, 10-HDA Level in Head, and Gene Expression in MG

**Figure 10:**
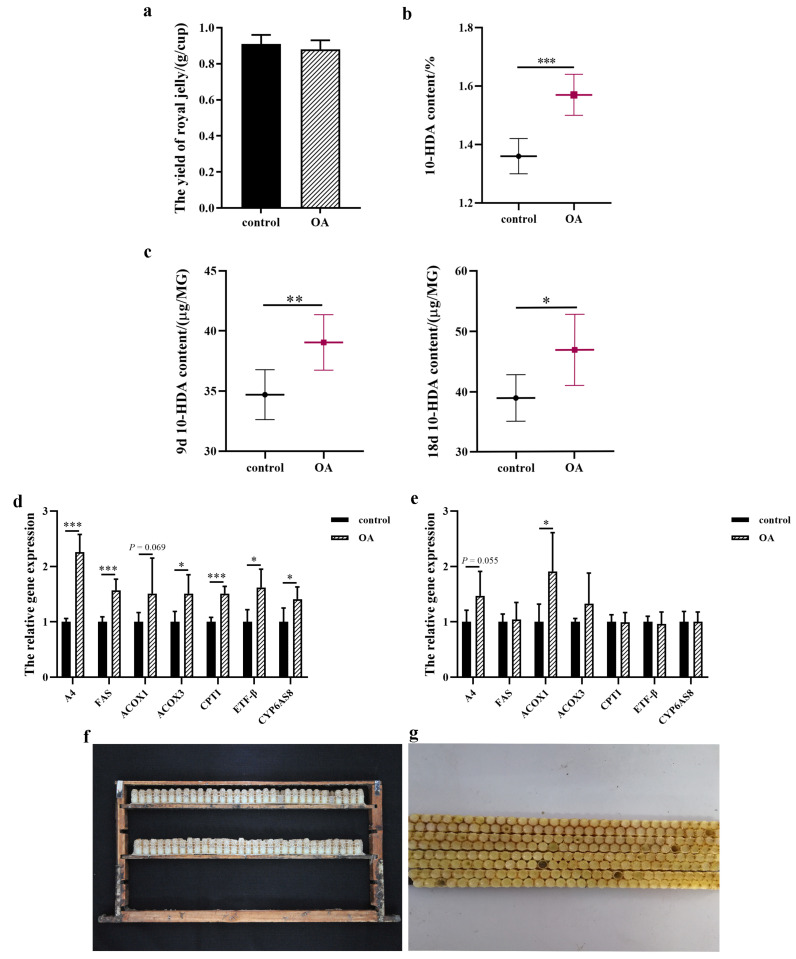
Royal jelly production in the two experimental groups (**a**). 10-HDA content in royal jelly collected from the two experimental groups (**b**). 10-HDA content in heads of 9- and 18-day-old worker bees in the two experimental groups (**c**). Relative mRNA expression levels of genes involved in 10-HDA biosynthesis in 9-day-old (**d**) and 18-day-old (**e**) worker bees. Values are expressed as means ± SEM (*n* = 8). (**f**) A royal jelly frame containing plastic queen cells with wax caps. (**g**) Wax caps removed and royal jelly left in the queen cell cups. * *p* < 0.05, ** *p* < 0.01, and *** *p* < 0.001.

RJ samples from all groups were thick and milky in appearance and slightly yellowish in color. There was no difference in RJ production between the two groups (Figure 10a). RJ samples collected from the bee colonies fed with the 8% OA diet contained higher levels of 10-HDA (1.57 ± 0.07%) than those fed with the control diet (1.36 ± 0.06%), an almost 1.15-fold difference (Figure 10b). Compared with the control group, feeding an 8% OA diet increased the 10-HDA content in the heads of 9- and 18-day-old worker bees by 1.13- and 1.20-fold, respectively (Figure 10c). Further, gene expression levels of *A4*, *FAS*, *ACOX3*, *CPT1*, *ETF-β*, and *CYP6AS8* were significantly increased in MGs of nine-day-old worker bees after OA supplementation (Figure 10d). OA supplementation had little effect on the expression of these genes in MGs from 18-day-old worker bees, except that there was increased expression of *ACOX1* (Figure 10e).

### 2.8. Determination of Lipid Composition in the Heads of Nine-Day-Old Worker Bees

**Figure 11:**
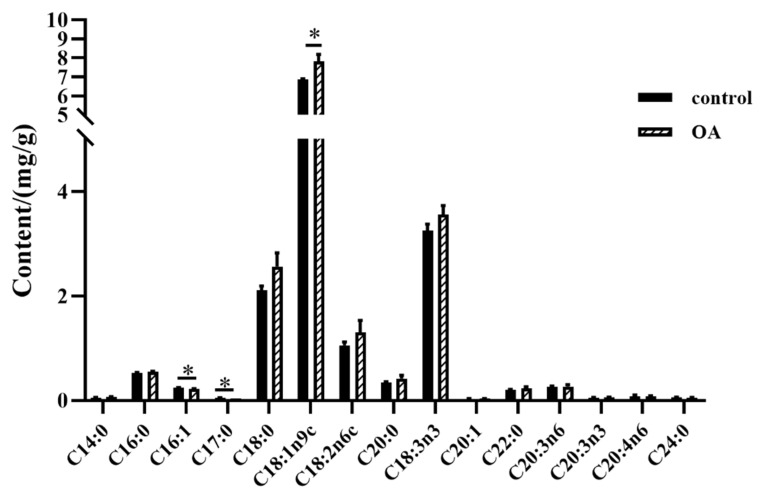
Fatty acid composition of the heads of 9-day-old worker bees. Values are expressed as means ± SEM (*n* = 3). * *p* < 0.05.

Fatty acids, as units of membrane phospholipids and a repository for lipids, play multiple functions, including influencing the formation and dynamic characteristics of biomembranes and serving as fuels for energy supply in mollusks [32]. Oleic acid (C18:1n9c), linolenic acid (C18:3n3), and stearic acid (C18:0) were the three most abundant fatty acids in worker bee heads (Figure 11). Dietary supplementation with 8% OA increased the OA content compared with controls, while levels of linolenic and stearic acids showed a tendency to increase (Figure 11). In addition, palmitoleic acid (C16:1) and margaric acid (C17:0) content decreased after feeding with an 8% OA diet (Figure 11), while myristic acid (C14:0) had a tendency to increase. Fatty acid analysis of bee samples showed that the amount of lipids and the omega-6:3 ratio generally reflected the composition of their diet [33].

## 3. Discussion

### 3.1. OA Induces 10-HDA Biosynthesis in Worker Bee MG in a Concentration-Dependent Manner

The compound 10-HDA is the major component of the worker bee MG [34,35]. It serves multiple purposes, including food preservation and nutrition for larvae [21]. Additionally, it also functions in determining the queen in larvae and regulating of honey bee behavior [36,37]. Through the use of stable isotope labeling, Plettner et al. [38] conducted a study that demonstrated the synthesis of 10-HDA from stearic acid in the species *A. mellifera*. This synthesis occurs through de novo fatty acid synthesis, where stearic acid is hydroxylated at the ω-position. The 18-carbon hydroxy acid is then shortened by β-oxidation, primarily resulting in a 10-carbon chain. It is likely that the double bond is introduced into the shortened ω-hydroxy acid chain by a desaturase [39].

Many insects can synthesize fatty acids de novo from acetyl-CoA precursors via the activity of FAS [40,41]. FAS is found in high abundance in workers producing high levels of 10-HDA, suggesting that the process of generating the stearic acid precursor of 10-HDA utilizes the same pathway as that for fatty acid synthesis [42]. Apolipophorin-III is an important lipid transport protein in insects [43] that has high affinity for PL and diacylglycerol surfaces [44]. In this study, compared with controls, expression levels of the *A4* and *FAS* genes increased in the 8% OA group and the 2% and 8% OA groups, respectively, suggesting that lipid synthesis and transport were intensive in bees fed the 8% OA diet.

In *Apis mellifera*, the P450 enzymes have primarily been linked to the process of detoxifying mycotoxins [45], flavonoids, acaricides, and insecticides [46]. Furthermore, P450 enzymes have been implicated in the synthesis of pheromones within the mandibular glands of honey bees [24]. CYP6AS8 plays a crucial role in the synthesis of mandibular acids in honey bee workers and is likely responsible for the hydroxylation of steric acid [47]. The downregulation of CYP6AS8 leads to a significant decrease in the content of 10-HDA, indicating that CYP6AS8 is essential for the biosynthesis of 10-HDA [47]. The vital role of CYP6AS8 in the hydroxylation processes involved in 10-HDA biosynthesis in worker bees has been extensively documented by Fujita et al. [48] and Wu et al. [24]. In our study, treatment with OA resulted in an increase in the expression of the *CYP6AS8* gene, suggesting that it promotes the hydroxylation of steric acid. In the mitochondria, the initial step of this process involves the uptake of acyl-CoA by CPT, which regulates the transport of fatty acids from the cytosol to the mitochondrial intermembrane space [49]. In the peroxisomal system, the ACOX enzymes catalyze the first step, controlling the flow through the pathway [50]. ETF-β acts as a specific electron acceptor for several mitochondrial dehydrogenases, including medium-chain acyl-CoA dehydrogenase and long-chain acyl-CoA dehydrogenase, which are involved in fatty acid β-oxidation [42]. Knocking down the expression of electron transfer flavoprotein subunit beta (ETF-β), a protein associated with fatty acid metabolism, resulted in a decrease in 10-HDA production in worker bees, indicating that ETF-β is necessary for the biosynthesis of 10-HDA [42]. In this study, different OA concentrations exerted varying influence on expression of the above genes, indicating vigorous 10-HDA secretion in insects fed with OA, particularly in the 8% OA group.

### 3.2. Species-Selective Remodeling of TAGs in MG Induced by 8% OA

As molecules essential for life, lipids play structural roles in cell membranes, provide resources for energy metabolism, and work as messengers in intracellular and intercellular signal communications [51]. In honey bees, lipids are important primarily as a source of energy, with some lipid components involved in the synthesis of reserve fat, glycogen, and cell membrane structures [52]. Further, lipid components—such as fatty acids and sterols—are important in honey bee development, nutrition, and reproduction. Lipidomics serves as a robust methodology for assessing alterations in lipid levels within organisms [53]. In the present study, we conducted lipidomics analysis using UPLC-Q-Exactive Orbitrap/MS, which facilitates identification of lipid components and characterization of differences in the MG of bees fed different diets. PC and TAG were the main lipids in the mandibular gland, consistent with our previous research [54]. TAGs are the predominant form of lipids in insects, functioning as energy storage molecules during non-feeding periods and long-distance flight [55,56] and as precursors for other molecules, such as semiochemicals [31]. As important neutral lipids, TAGs contain a glycerol backbone and three FA chains [51]. The fatty acids produced by triglyceride hydrolysis can be used as substrates for synthesis of 10-HDA in the worker bee MG. Although OA supplementation did not alter the overall abundance of TAGs, the levels of many types of TAG changed significantly, and the FA chain composition of increased TAGs was, interestingly, mainly C18:1. Through lipidomics analysis, we found that 10 types of TAG increased in response to OA treatment, with the abundance of TAG (18:1-18:1-18:1) the highest, followed by TAG (18:0-18:1-18:1).

Transcriptome analysis showed higher *Gpat* and *Gk* expression in the OA group than in controls. De novo synthesis of TAG in mammalian cells begins with the acylation of glycerol-3-phosphate, catalyzed by glycerol-3-phosphate acyltransferase (GPAT), which is believed to be a rate-limiting step [57]. As occurs in many other lipid metabolic reactions, this activity is catalyzed by several GPAT isoforms. Four different genes encode GPAT isoforms 1–4, which differ in tissue expression pattern and subcellular localization [58]. Glycerol is an organic compound that can be utilized as an alternative source of carbon by various organisms. Cells can assimilate glycerol via the phosphorylative catabolic pathway, which is catalyzed by glycerol kinase (GK), and glycerol-3-phosphate (G3P) is formed [59]. GK is conserved among prokaryotes and eukaryotes [60]. The increased transcription levels of *Gpat* and *Gk* observed in this study may have been responsible for the higher TAG (18:1-18:1-18:1) and TAG (18:0-18:1-18:1) levels detected, as the MG preferentially synthesized these two kinds of lipid. Lipase is an enzyme that breaks down triglycerides into free fatty acids and glycerol, and LOC727173, encoding triacylglycerol lipase, increased expression in the OA group, suggesting that stored triglycerides were hydrolyzed to free fatty acids. Mobilization of stored TAG both serves as an energy source and provides free fatty acid (FFA) for membrane lipid synthesis, facilitating maintenance of membrane homeostasis during fly maturation and aging [61]. Based on our results, we speculate that OA in the diet was directly used in the MG in TAG (18:1-18:1-18:1) synthesis, which was stored in the MG. Some of the OA produced by hydrolysis of stored TAG (18:1-18:1-18:1) was utilized as a precursor to synthesize 10-HDA; however, the process involved in conversion of OA into stearic acid requires further exploration.

### 3.3. 8% OA Increased 10-HDA Levels in RJ

In bee colonies, the production of jelly by nurses is essential for feeding the larvae and the queen. This jelly is derived from their hypopharyngeal glands and MGs. During the first 8 days of development, the larvae consume a significant amount of food before the cells are sealed to allow for metamorphosis [21]. It has been observed that inadequate nutrition in the colony hinders the full development of nurse bee head glands, increases larval cannibalism by nurse bees, and leads to a decrease in the number of sealed brood cells [62,63]. Consequently, the number of sealed brood cells is often used as an indicator of successful brood-rearing. Numerous studies have investigated the impact of lipid content on the development of the sealed brood. For instance, it has been found that nurse bees fed a diet with a high lipid percentage (8%) were able to rear a greater proportion of young larvae to the sealed stage compared to the control group that received an artificial diet with negligible lipid content [33]. Similarly, Herbert et al. [64] demonstrated that a colony was unable to successfully rear larvae to produce sealed broods when fed a fat-free pollen substitute. Furthermore, their research indicated that bees with diets containing 2–4% lipids had a slightly larger total sealed brood area compared to those receiving 6–8% lipids [64]. In our study, addition of 8% OA had no effect on sealed brood development. This may be because the lipid in the control artificial diet feed was adequate to meet the needs for development of nurse bee head glands. Alternatively, our findings may reflect that short-term feeding of RJ with higher 10-HDA did not promote queen fecundity.

Numerous factors influence the RJ content of 10-HDA, and the 10-HDA content in commercial RJ varies considerably, at between 1.4% and 3.4% of wet matter [15,19,65,66,67], and can be affected by geographical origin [19,67], bee strain [68], and harvest time [69], among other factors. There are few reports about the effect of diet on 10-HDA content in RJ. Pattamayutanon et al. [70] reported that RJ samples from bee colonies fed by pollen from bitter bush and coffee possessed higher 10-HDA levels than those collected from bee colonies fed by tea pollen. In our study, diets supplemented with 8% OA led to increased 10-HDA content in RJ. Further, 10-HDA levels were higher in 9- and 18-day-old worker bee heads than in those of controls, suggesting that in nurse and forager bees, OA promotes 10-HDA secretion in the MG; however, OA did not significantly affect expression of genes involved in 10-HDA synthesis in the MG of 18-day-old worker bees, possibly because OA treatment led to an earlier peak in 10-HDA secretion. Further, forager bees maintained higher 10-HDA levels in the head, possibly because 10-HDA secreted earlier was stored as a reservoir.

In conclusion, our integrated results indicate that supplementation with 8% OA led to increased expression of genes involved in 10-HDA biosynthesis, promoted its secretion, and eventually resulted in increased 10-HDA content in RJ. Further, OA supplementation induced gene expression programs involved in glycerolipid metabolism, accompanied by a marked increase in the abundance of TAG (18:1-18:1-18:1) and TAG (18:0-18:1-18:1). The acyl chain compositions of these two TAGs are 18:0 and 18:1, which may be used as substrates for 10-HDA synthesis.

## 4. Materials and Methods

### 4.1. Trial 1: Supplementation of Honey Bee Diet with Different Levels of OA

All experimental honeybee colonies were maintained at the Shandong Agricultural University apiary in Tai’an City, China, from April to July 2019. At the start of the trial, frames with sealed worker broods from natural colonies were put in an incubator at 34.5 ± 1 °C and 70 ± 5% relative humidity (RH). When the new workers emerged, they were pooled and transferred into cages (23 cm × 9.5 cm × 5 cm, length × width × height; 60 bees per cage). A total of 1200 newly emerged worker bees were randomly divided into five groups (four replicates per group with 60 bees per replicate): a control group receiving artificial diet and experimental groups receiving 2%, 4%, 6%, and 8% OA addition. The addition of 2, 4, 6, and 8% OA to the diet was based upon powder mass percent. The ingredients of powder are shown in Supplementary Table S1. The procedure of artificial diet preparation was as follows: the sugar was added into the powder and well mixed, then the water was provided into the mixture. Finally, the preparation was carried out according to the ratio of 1 (powder):1.5 (sugar):0.68 (water). The diet was sealed and fermented for 12 h, then stored at 4 °C. When feeding, artificial diets were added on a coverslip (2.4 cm × 2.4 cm) on the bottom of each cage, changed every two days to ensure the freshness. During the trail, the bees had ad libitum access to water and 50% (*w*/*w*) sugar solution using a microwell plate. Cages were checked daily to adjust sugar solution and artificial diet supply, and dead bees were removed. The fatty acid composition of powder was measured by a commercial company (Weifang Hairun Huachen Testing Technology Co., Ltd., Weifang, China), and detected fatty acids are shown in Supplementary Table S2. All cages were reared in an insect incubator at 32 °C and 50% relative humidity for 9 days. In each cage, 40 bees were collected for mandibular glands, 20 pairs of them as one sample for analysis of gene expression; 10 bees were collected for the heads and 5 pairs as one sample for measurement of 10-HDA content. All samples were stored at −80 °C until analysis.

MGs were collected according to previous reports [54,71,72] with modification. Honey bees were immobilized by freezing at −20 °C, then pinned on wax plates using an insect needle. Under a stereoscope, we used dissecting forceps to carefully separate the mandibular glands from the bee’s head. The MG together with the mandible were removed out of the head capsule and placed on a slide containing cold insect Ringer’s solution (pH 7.4; 6.4 mL 5 M NaCl, 3.75 mL 0.1 M CaCl_2_, and 1.25 mL 1 M KCl). Then, the complete MG was separated from the mandible, frozen in liquid nitrogen, and placed at −80 °C until analysis. All operations were conducted gently and with care.

### 4.2. Trial 2: Supplementation of Honey Bee Diet with 8% Level of OA

According to the results of trial 1, we chose to study the effects of 8% OA supplementation on changes of lipids in the MG. A total of 2200 newly emerged bees from five colonies were randomly divided into two groups, containing 11 cages per group with 100 bees per cage. Diet preparation and feeding procedures were similar to those for trial 1. On the 9th day of feeding, MGs were collected from bees and used for transcriptome analysis, including three samples (60 bees from one cage pooled as one sample) per group, and lipidomics analysis, including four samples (180 bees from two cages pooled as one sample) per group. The samples for transcriptome and lipidomics analysis were stored at −80 °C until analysis. Twenty pairs of MGs from four cages in each group were collected and prepared for transmission electron microscope observation.

### 4.3. Trial 3: Supplementation of Colony with 8% Level of OA

Ten healthy colonies with no sign of disease were used in this study, located at the Shandong Agricultural University apiary in Tai’an City, China. The experiment was carried out in June and August of 2020. At the beginning of the study, combs with stored natural pollen were replaced with empty ones, and sealed brood combs without workers were exchanged among colonies to ensure a similar number of workers and broods among these colonies (following a visual inspection). Five colonies were fed with an artificial diet (control group), and the remaining five colonies received an 8% OA addition diet (OA group). The treatments were arranged as a complete randomized design. All colonies were adapted to an artificial diet for 8 days first; then, the experiment was conducted for 60 days. Pollen traps were fitted to hives at all stages in order to prevent importing of pollen. All the diets were processed into patties according to the ratio of 1 (powder):1.5 (sugar):0.68 (water). After sealed fermentation for 12 h, the patty (500 g) for each colony was then flattened and placed on the top of the frames over the brood cluster. In order to keep the freshness, the patties were changed every three days. The bees had access to water and the patty ad libitum. At 21 days (to ensure that nurse bees were reared while exposed to the OA treatment) after the adaptation period, royal jelly was collected and newly emerged bees were marked on the thorax. We collected the mandibular gland (20 pairs of mandibular glands pooled as one sample) for analysis of gene expression and head (10 heads pooled as one sample) of 9-day-old and 18-day-old worker bees for measurement of 10-HDA level, and froze the samples at −80 °C until detection.

### 4.4. Measurement of the Average Food Intake of Worker Bees Reared in Cages

Food and feeding plates were weighed together before being given to bees. To maintain the palatability and freshness of the diet, food was changed every two days and the remaining food weighed with the plate before being replaced with fresh food. Final feed consumption was calculated as the AFI per bee.

### 4.5. Measurement of 10-HDA Content in Head

Content of 10-HDA in the head of worker bees was determined by high-performance liquid chromatography (HPLC), as previously described [54,73]. Heads of worker bees were placed in a centrifuge tube containing 1 mL of methanol (chromatography grade) and steel balls, then thoroughly homogenized by tissue grinders. After that, the homogenate was transferred to a 10 mL volumetric flask, and the steel balls were washed three times using methanol solution. Then, 1 mL of methyl-4-hydroxybenzoate solution (final concentration, 0.653 mg/mL) was added to the volumetric flask as an internal standard. The sample volume was made up to 10 mL with methanol. After ultrasonication for 15 min, analyte was further extracted and then filtered through 0.45 μm microporous filters. Aliquots (20 μL) of sample solution were injected into an Agilent Technologies 1200 series liquid chromatography system. A standard calibration curve was constructed according to a previous report [54]. The Agilent Technologies 1200 series high-performance liquid chromatography system equipped with an Agilent diode array detector and a reversed phase C18 column (4.6 × 250 mm, 5 μm) (Agilent Technologies Inc., Santa Clara, CA, USA). was applied. The mobile phase was composed of methanol/0.2% H_3_PO_4_ (pH 3) (*v*/*v* = 55:45) at a flow rate of 1 mL/min. Column temperature was adjusted to 25 °C, and the detector was adjusted to 210 nm.

### 4.6. Measurement of Gene Expression in Mandibular Gland

Gene expression in MGs was assessed byqRT-PCR with SYBR Green I labeling, as in our previous research [54]. MGs from 20 workers bee were pooled as one sample. Total RNA was extracted using an RNA extraction kit (TIANGEN, Beijing, China) according to the manufacturer’s instructions. The quality and quantity of RNA were determined by agarose gel electrophoresis and a biophotometer (Eppendorf, Germany), respectively. We used a PrimeScript RT reagent kit (Takara Bio, Kyoto, Japan) to reverse transcribe RNA into cDNA. qRT-PCR was conducted using a 7500 Real-time PCR System (Applied Biosystems, Foster, CA, USA). Each RT reaction contained 0.2 μmol/L of each primer and SYBR green master mix and cDNA template as 20 μL reactions (Takara, Dalian, Liaoning, China). The primer sequences are provided in Supplementary Table S3. The gene encoding *rp49* was used as a reference housekeeping gene [74], and the results were verified using β-actin levels. The relative amount of mRNA was calculated according to the method described by reference [75].

### 4.7. RNA Sequencing and Non-Targeted Lipidomics Analysis of Mandibular Gland

Total RNA was extracted from 60 pairs of MGs using Trizol Reagent (Invitrogen, Carlsbad, CA, USA). The integrity and quantity of RNA were detected by an Agilent 2100 biological analyzer. Preparing Illumina RNA-seq libraries and performing the paired-end sequencing (300 ± 50 bp) on an Illumina Hiseq 4000 (LC Sceiences, Houston, TX, USA) were completed by a vendor (Beijing Novogene Technology Co., Ltd., Beijing, China). Clean reads were mapped to the *A. mellifera* reference genome in the NCBI database (Amel_HAv3.1) using Hisat2. Gene expression levels were normalized as the number of reads per kilobase of exon region per million mapped reads (FPKM). DESeq2 was used for differential expression analysis. Adjusted *p*-values were adjusted using the Benjamini Hochberg approach to control the false discovery rate (FDR). Differentially expressed genes (DEGs) were defined with adjusted *p*-values < 0.05 and |log2 (fold-change)| > 0. Analysis of pathways enriched for DEGs was performed using the clusterProfiler R package, based on the KEGG database. Nine randomly selected DEGs identified by transcriptome sequencing analysis were used for RNA-seq validation by qRT-PCR. Specific primers are shown in Supplementary Table S3.

Frozen MG samples were homogenized in 750 µL ice-cold methanol, and then methyl tert-butyl ether (MTBE; 2.5 mL) was added. After incubation for 1 h at room temperature, 0.625 mL of MS-grade water was added to separate the organic phase. The samples were centrifuged at 1000× *g* for 10 min after 10 min incubation at room temperature. The upper (organic) phase was collected, the lower phase was re-extracted with 1 mL of the solvent mixture (MTBE/methanol/water (10:3:2.5, *v*/*v*/*v*)), and the upper phase collected. Organic phases were dried and dissolved in 100 μL isopropanol for analysis by LC-MS/MS. Chromatographic separation was performed using a Thermo Vanquish UHPLC system (Thermo Fisher, Karlsruhe, Germany). Mass spectrometric detection was performed on an Orbitrap Q Exactive^TM^ HF mass spectrometer (Thermo Fisher, Karlsruhe, Germany). Samples were injected onto a Thermo Accucore C30 column (150 × 2.1 mm, 2.6 μm) using a 20 min linear gradient at a flow rate of 0.35 mL/min. The column temperature was set at 40 °C. Mobile phase buffer A was acetonitrile/water (6/4) with 10 mM ammonium acetate and 0.1% formic acid, whereas buffer B was acetonitrile/isopropanol (1/9) with 10 mM ammonium acetate and 0.1% formic acid. Q ExactiveTM HF mass spectrometer was operated in positive [negative] polarity mode with sheath gas: 20 arbitrary units; sweep gas: 1 arbitrary unit; auxiliary gas rate: 5 [7]; spray voltage: 3 kV; capillary temperature: 350 °C; heater temperature: 400 °C; S-Lens RF level: 50; resolving power (full scan): 120,000; scan range: 114–1700 *m*/*z*; automatic gain control target: 1 × 10^6^; resolving power (MS^2^): 30,000 (Top20); normalized collision energy: 22; 24; 28 [20; 24; 28]; injection time: 100 ms, isolation window: 1 *m*/*z*; automatic gain control target (MS2): 1 × 10^5^; dynamic exclusion: 15 s. All operations were completed by Beijing Novogene Technology Co., Ltd. (Beijing, China). Raw data files generated by UHPLC-MS/MS were processed using Compound Discoverer 3.1 (CD3.1, Thermo Fisher). After that, peaks were then matched against the Lipidmaps, Lipidblast, and HMDB databases. Statistical analyses were performed using the statistical software R (R version R-3.4.3), Python (Python 2.7.6 version), and CentOS (CentOS release 6.6), When data were not normally distributed, normal transformations were attempted using the area normalization method. PCA and PLS-DA were performed using metaX (a flexible and comprehensive software for processing metabolomics data). Univariate analysis (*t*-test) was applied to calculate statistical significance (*p*-value). Metabolites with VIP > 1 and FC > 1.2 or FC < 0.833 and *p*-value < 0.05 were considered to be different between samples.

### 4.8. Transmission Electron Microscopy

The MGs collected from worker bees were immediately fixed in 2.5% glutaraldehyde in 0.1 M phosphate buffer (PBS, pH 7.2) for 24 h at 4 °C [54]. After rinsing twice with PBS, the samples were fixed in 1% osmium tetroxide. After rinsing twice with PBS, the samples were dehydrated in a series of ethanol solutions (45% to 100%) and embedded in Araldite. Semi-thin sections (2 μm) were cut using a Leica Ultracut ultramicrotome (Leica, Wetzlar, Germany), stained with methylene blue and thionine, and viewed using an Olympus BX-51 microscope. Ultrathin sections (80 nm) were contrasted with uranyl acetate and lead citrate and then examined and photographed using a JEM-1230 transmission electron microscope.

### 4.9. Measurement of Bee Population, Sealed Brood Development, and Consumption

The population of worker bees was recorded every 11 days by estimating the area of frame covered with bees (one frame with two sides fully covered by single-layer bees was designated as 1-frame. If one frame partly half covered by single-layer bees was designated as 0.5-frame). Measurements of all frames with worker bees were summed for each colony. The amount of sealed brood was estimated using the same method. Bee population and sealed brood development were recorded for three times to judge the effect of long-term feeding with oleic acid on the bee colony. At the beginning, all bee colonies had their own queen, and the population of worker bees was around 11 frames and amount of sealed brood was around 3 frames in each colony. During the experimental period, food consumption was recorded and calculated as total mixture and powder, respectively.

### 4.10. Measurement of RJ Production

A frame contained 126 plastic queen cell cups fixed on two strips of wood bars. For RJ collection, the frame was placed into each colony. The cups were moistened with a small amount of royal jelly in advance to improve the acceptance rate of larvae. The young worker larvae (~24 h old) were grafted into queen cell cups, and then the frames were put back into the colonies. About 68–72 h later, we took out the frames from the colonies for RJ collection, and all drawn queen cells were counted and removed for weighing and sampling. After removing both the wax caps at the top of the plastic queen cell cups and the larvae in the cells, the RJ was then collected and measured by an electronic scale (PL1002E/02, METTLER TOLEDO, with a readability of 0.01 g). In total, three rounds of grafting were performed consecutively. All the colonies were rewarded with equal amounts of sugar solution (about 1 kg) following grafting to encourage queen rearing. All the colonies were inspected between grafts to remove any naturally produced queen cells. The collected RJ samples were kept at −20 °C until analysis.

### 4.11. Measurement of 10-HDA Concentration in RJ

We measured the 10-HDA concentration in RJ according to standard Chinese methods (GB/9697-2008) with some modifications. Royal jelly samples were thawed at room temperature. An aliquot of 0.5 g homogenated RJ (mixture of three rounds) was weighed in a centrifuge tube, dissolved by adding 1 mL of 0.03 M HCl and 2 mL redistilled water, and vortexed for 30 s to dissolve the sample. Then, all liquid was transferred to a 50 mL volumetric flask, 10 mL of methyl-4-hydroxybenzoate solution (0.65 mg/mL) was added, and finally the volume was compensated with ethanol. The analyte was extracted by ultra sonification for 15 min. The solution was filtered through a 0.22 μm membrane. The volume of the 20 μL sample solution was injected in the Agilent Technologies 1200 series liquid chromatographic system equipped with an Agilent diode array detector and a reversed phase C18 column (4.6 × 250 mm, 5 μm)(Agilent Technologies Inc., Santa Clara, CA, USA). A standard calibration curve was constructed as mentioned above. The mobile phase was composed of methanol/0.03 M HCl/H_2_O (*v*/*v*/*v* = 55/10/35) at a flow rate of 1 mL/min. The column temperature was adjusted to 35 °C during the experiment, and the detector was adjusted to 210 nm.

### 4.12. Measurement of Fatty Acid Composition

The fatty acids of worker bees’ heads (fifty heads pooled as one sample) were extracted with benzene/petroleum ether (1:1, *v/v*) for 12 h in a glass tube with stopper. Then, 2 mL esterifying agent (0.4 mol/L potassium hydroxide methanol solution) was added to the glass tube for 15 min. The distilled water was added slowly to the tube. When the liquid level reached the mouth of the bottle, 200 mL of absolute ethanol was added. The supernatant was transferred to a centrifuge tube and centrifuged at 12,000 rmp for 5 min. A gas chromatograph (GC-2010, Shimadzu Co., Kyoto, Japan) equipped with a flame ionization detector was applied to separate fatty acid methyl esters. The sample (1 µL) was injected into the chamber and separated on a capillary column (DB-23, 30 m × 0.25 mm × 0.25 µm) by nitrogen as the carrier gas. Fatty acid identification was achieved by comparing retention times with a standard mix (CDDE-GLC-746B-100MG, Nu-Chek Prep., Elysian, MN, USA).

### 4.13. Statistical Analysis

Statistical analysis was carried out by one-way ANOVA (version 8.02; SAS Institute Inc., Cary, NC, USA), followed by a Tukey’s post hoc test, to determine the statistical significance of the data in Trial 1. The two groups were compared via *t*-test in Trial 2 and 3. Data are presented as mean ± SEM. *p* values < 0.05 were considered statistically significant.

## Figures and Tables

**Table 1:** Average feed intake of bees reared in cages.

	OA Concentration					
	Control	2%	4%	6%	8%	*p*
0–2 days	20.42 ± 2.14	22.36 ± 1.70	17.02 ± 1.79	16.70 ± 1.31	12.84 ± 0.37 *	0.0068
2–4 days	32.63 ± 1.41	27.29 ± 1.25 *	28.85 ± 1.32	27.75 ± 1.55	27.34 ± 2.19	0.1427
4–6 days	21.79 ± 3.02	20.49 ± 1.80	19.94 ± 2.23	24.01 ± 2.93	24.10 ± 1.98	0.6487
6–8 days	10.56 ± 0.69	10.64 ± 0.28	11.69 ± 0.59	12.73 ± 0.50 *	13.96 ± 1.57	0.0587

* *p* < 0.05 compared with the control group. Units, mg/bee; *n* = 4.

**Table 2:** Percentage of major TAG species in the control and OA-supplemented groups.

Lipid Species	Lipid Percentage in Each Group (%)	
	Control	OA
TAG (18:1-18:1-18:1)	10.82	16.42
TG (16:1 (9Z)/18:1 (9Z)/18:1 (9Z)) [iso3]	10.49	11.04
TAG (16:0-18:1-18:1)	10.30	9.01
TG (16:1 (9Z)/16:1 (9Z)/18:0) [iso3]	7.28	5.76
TAG (16:0-18:1-18:3)	6.86	6.20
TAG (18:1-18:2-18:2)	5.59	6.84
total	51.34	55.27

**Table 3:** Major characteristics of total raw reads for each library generated by RNA-Seq.

Sample	Raw Reads	Clean Reads	Clean Base Number	Total Mapped	Mapped Ratio (%)	Q20 (%)	Q30 (%)	GC Content (%)
OA-1	42,754,450	41,828,552	6.27 G	40,090,855	95.85	97.4	92.89	34.98
OA-2	41,140,222	40,306,446	6.05 G	38,489,735	95.49	97.02	92.07	34.05
OA-3	45,942,544	45,091,526	6.76 G	42,179,507	93.54	97.28	92.66	34.88
control-1	46,316,898	45,100,268	6.77 G	42,839,085	94.99	97.96	93.86	33.35
control-2	49,256,756	48,479,402	7.27 G	38,760,127	79.95	97.81	93.65	34.6
control-3	46,511,288	45,833,138	6.87 G	40,997,561	89.45	97.88	93.78	34.7

## Data Availability

All data are contained within the manuscript, including Supplemental Information.

## Supplementary Materials

The supporting information can be downloaded at: https://www.mdpi.com/article/10.3390/ijms241713361/s1.
