# Supplementary material for: Oleic Acid Promotes the Biosynthesis of 10-Hydroxy-2-decenoic Acid via Species-Selective Remodeling of TAGs in Apis mellifera ligustica
Source: Int J Mol Sci. 2023 Aug 29;24(17):13361. doi: 10.3390/ijms241713361 (PMC10487919; doi:10.3390/ijms241713361)

**Supplementary Figure S1.** Expression levels of nine selected differentially expressed genes (DEGs) normalized to rp49 of *Apis mellifera* mandibular gland. Notes: Note:* indicates the significance levels at $p < 0.05$. Data are presented as mean ± SEM (n = 3).

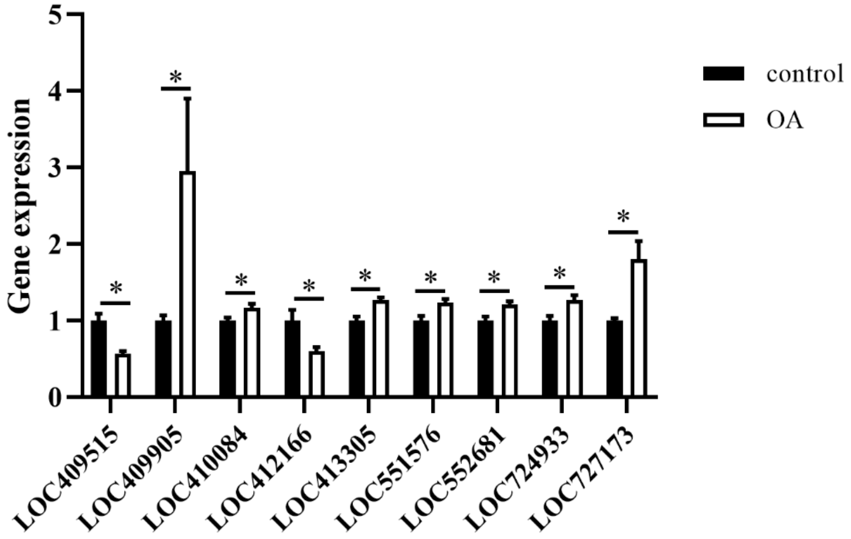

**Supplementary Figures S2.** The cluster heatmap of different lipids of the two treatment groups. (**a**) in negative ion mode; (**b**) in positive ion mode.

**(a)**

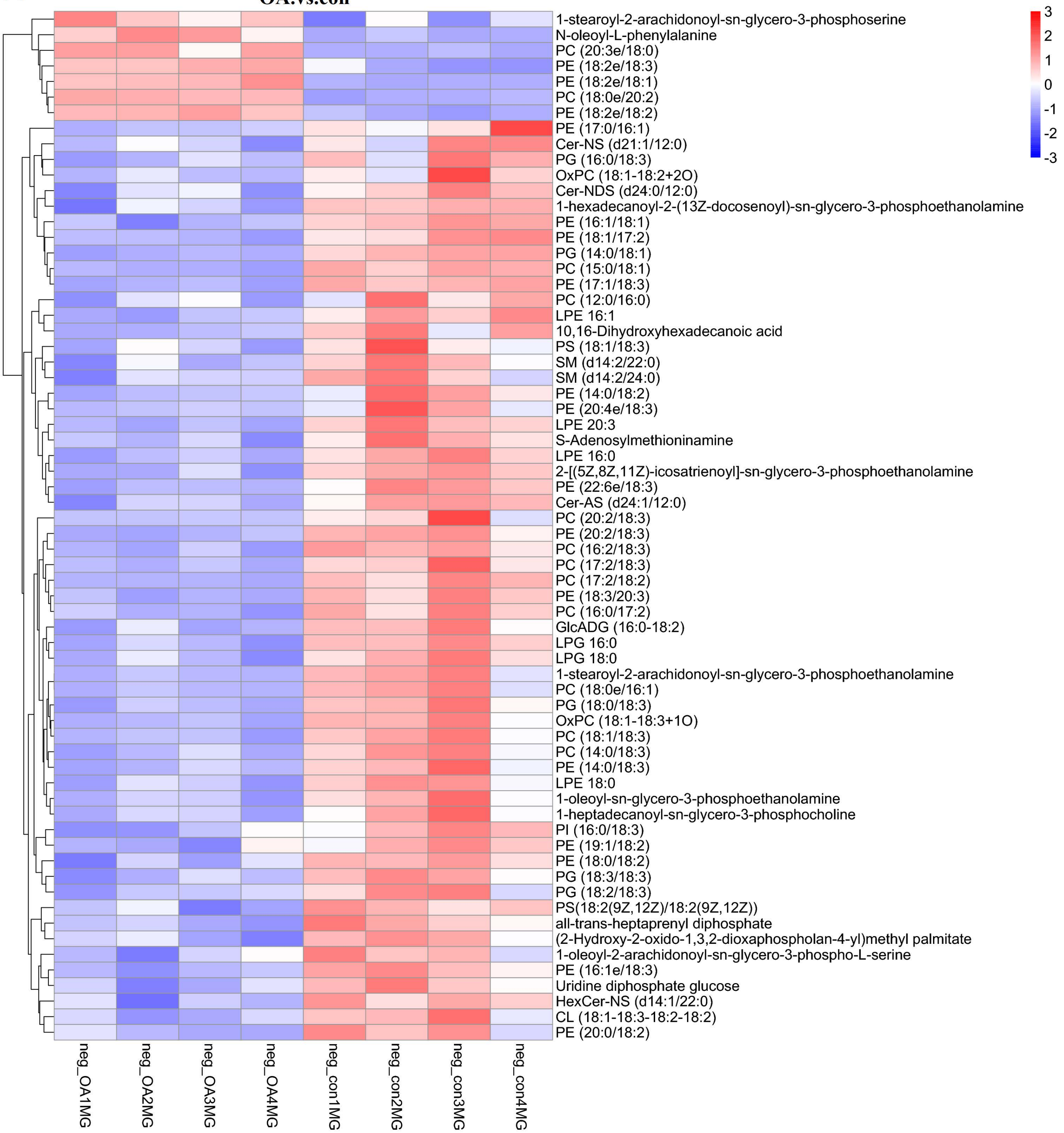

(b)

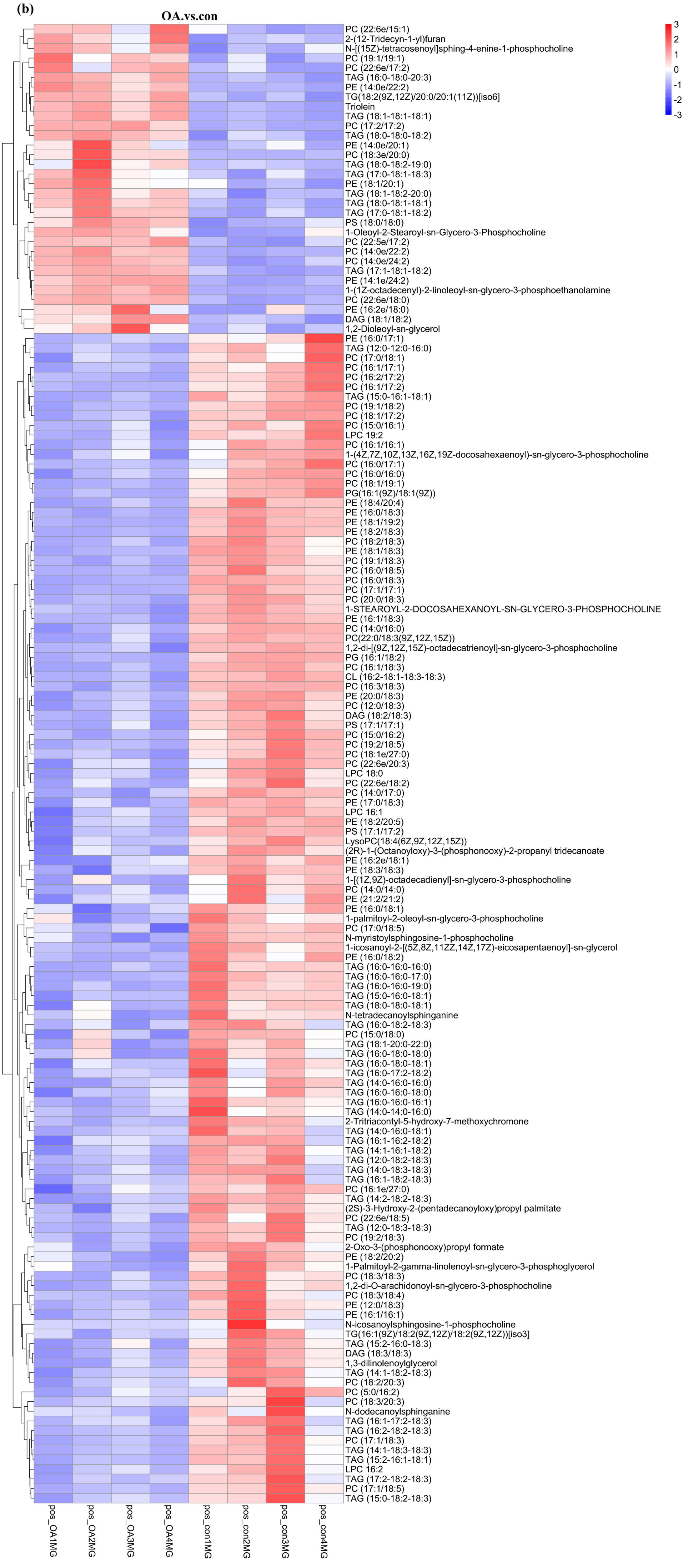
OA.vs.con
3
2
1
0
-1
-2
-3
PC (22:6e/15:1)
2-(12-Tridecyn-1-yl)furan
N-[(15Z)-tetracosenoyl]sphing-4-enine-1-phosphocholine
PC (19:1/19:1)
PC (22:6e/17:2)
TAG (16:0-18:0-20:3)
PE (14:0e/22:2)
TG(18:2(9Z,12Z)/20:0/20:1(11Z))[iso6]
Triolein
TAG (18:1-18:1-18:1)
PC (17:2/17:2)
TAG (18:0-18:0-18:2)
PE (14:0e/20:1)
PC (18:3e/20:0)
TAG (18:0-18:2-19:0)
TAG (17:0-18:1-18:3)
PE (18:1/20:1)
TAG (18:1-18:2-20:0)
TAG (18:0-18:1-18:1)
TAG (17:0-18:1-18:2)
PS (18:0/18:0)
1-Oleoyl-2-Stearoyl-sn-Glycero-3-Phosphocholine
PC (22:5e/17:2)
PC (14:0e/22:2)
PC (14:0e/24:2)
TAG (17:1-18:1-18:2)
PE (14:1e/24:2)
1-(1Z-octadecenyl)-2-linoleoyl-sn-glycero-3-phosphoethanolamine
PC (22:6e/18:0)
PE (16:2e/18:0)
DAG (18:1/18:2)
1,2-Dioleoyl-sn-glycerol
PE (16:0/17:1)
TAG (12:0-12:0-16:0)
PC (17:0/18:1)
PC (16:1/17:1)
PC (16:2/17:2)
PC (16:1/17:2)
TAG (15:0-16:1-18:1)
PC (19:1/18:2)
PC (18:1/17:2)
PC (15:0/16:1)
LPC 19:2
PC (16:1/16:1)
1-(4Z,7Z,10Z,13Z,16Z,19Z-docosahexaenoyl)-sn-glycero-3-phosphocholine
PC (16:0/17:1)
PC (16:0/16:0)
PC (18:1/19:1)
PG(16:1(9Z)/18:1(9Z))
PE (18:4/20:4)
PE (16:0/18:3)
PE (18:1/19:2)
PE (18:2/18:3)
PC (18:2/18:3)
PE (18:1/18:3)
PC (19:1/18:3)
PC (16:0/18:5)
PC (16:0/18:3)
PC (17:1/17:1)
PC (20:0/18:3)
1-STEAROYL-2-DOCOSAHEXANOYL-SN-GLYCERO-3-PHOSPHOCHOLINE
PE (16:1/18:3)
PC (14:0/16:0)
PC(22:0/18:3(9Z,12Z,15Z))
1,2-di-[(9Z,12Z,15Z)-octadecatrienoyl]-sn-glycero-3-phosphocholine
PG (16:1/18:2)
PC (16:1/18:3)
CL (16:2-18:1-18:3-18:3)
PC (16:3/18:3)
PE (20:0/18:3)
PC (12:0/18:3)
DAG (18:2/18:3)
PS (17:1/17:1)
PC (15:0/16:2)
PC (19:2/18:5)
PC (18:1e/27:0)
PC (22:6e/20:3)
LPC 18:0
PC (22:6e/18:2)
PC (14:0/17:0)
PE (17:0/18:3)
LPC 16:1
PE (18:2/20:5)
PS (17:1/17:2)
LysoPC(18:4(6Z,9Z,12Z,15Z))
(2R)-1-(Octanoyloxy)-3-(phosphonooxy)-2-propanyl tridecanoate
PE (16:2e/18:1)
PE (18:3/18:3)
1-[(1Z,9Z)-octadecadienyl]-sn-glycero-3-phosphocholine
PC (14:0/14:0)
PE (21:2/21:2)
PE (16:0/18:1)
1-palmitoyl-2-oleoyl-sn-glycero-3-phosphocholine
PC (17:0/18:5)
N-myristoylsphingosine-1-phosphocholine
1-icosanoyl-2-[(5Z,8Z,11ZZ,14Z,17Z)-eicosapentaenoyl]-sn-glycerol
PE (16:0/18:2)
TAG (16:0-16:0-16:0)
TAG (16:0-16:0-17:0)
TAG (16:0-16:0-19:0)
TAG (15:0-16:0-18:1)
TAG (18:0-18:0-18:1)
N-tetradecanoylsphinganine
TAG (16:0-18:2-18:3)
PC (15:0/18:0)
TAG (18:1-20:0-22:0)
TAG (16:0-18:0-18:0)
TAG (16:0-18:0-18:1)
TAG (16:0-17:2-18:2)
TAG (14:0-16:0-16:0)
TAG (16:0-16:0-18:0)
TAG (16:0-16:0-16:1)
TAG (14:0-14:0-16:0)
2-Tritriacontyl-5-hydroxy-7-methoxychromone
TAG (14:0-16:0-18:1)
TAG (16:1-16:2-18:2)
TAG (14:1-16:1-18:2)
TAG (12:0-18:2-18:3)
TAG (14:0-18:3-18:3)
TAG (16:1-18:2-18:3)
PC (16:1e/27:0)
TAG (14:2-18:2-18:3)
(2S)-3-Hydroxy-2-(pentadecanoyloxy)propyl palmitate
PC (22:6e/18:5)
TAG (12:0-18:3-18:3)
PC (19:2/18:3)
2-Oxo-3-(phosphonooxy)propyl formate
PE (18:2/20:2)
1-Palmitoyl-2-gamma-linolenoyl-sn-glycero-3-phosphoglycerol
PC (18:3/18:3)
1,2-di-O-arachidonoyl-sn-glycero-3-phosphocholine
PC (18:3/18:4)
PE (12:0/18:3)
PE (16:1/16:1)
N-icosanoylsphingosine-1-phosphocholine
TG(16:1(9Z)/18:2(9Z,12Z)/18:2(9Z,12Z))[iso3]
TAG (15:2-16:0-18:3)
DAG (18:3/18:3)
1,3-dilinolenoylglycerol
TAG (14:1-18:2-18:3)
PC (18:2/20:3)
PC (5:0/16:2)
PC (18:3/20:3)
N-dodecanoylsphinganine
TAG (16:1-17:2-18:3)
TAG (16:2-18:2-18:3)
PC (17:1/18:3)
TAG (14:1-18:3-18:3)
TAG (15:2-16:1-18:1)
LPC 16:2
TAG (17:2-18:2-18:3)
PC (17:1/18:5)
TAG (15:0-18:2-18:3)
pos_OA1MG
pos_OA2MG
pos_OA3MG
pos_OA4MG
pos_con1MG
pos_con2MG
pos_con3MG
pos_con4MG

**Supplementary Figure S3.** DEG distribution of the two treatment groups (**a**); cluster analysis of differentially expressed genes (**b**).

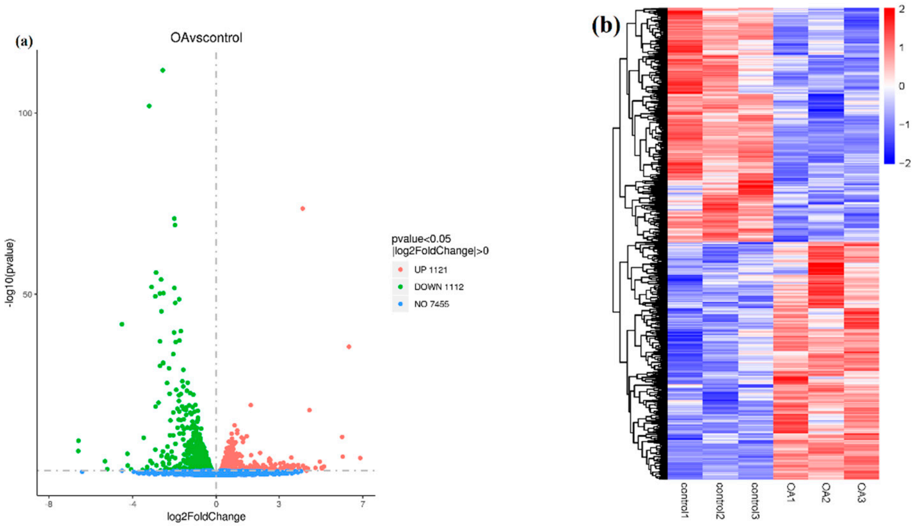

Supplement: Supplementary file 1 [file ijms-24-13361-s001.zip › Supplementary Figures S1-S3.pdf]
