# Supplementary material for: Oleic Acid Promotes the Biosynthesis of 10-Hydroxy-2-decenoic Acid via Species-Selective Remodeling of TAGs in Apis mellifera ligustica
Source: Int J Mol Sci. 2023 Aug 29;24(17):13361. doi: 10.3390/ijms241713361 (PMC10487919; doi:10.3390/ijms241713361)
Supplement: Supplementary file 1 [file ijms-24-13361-s001.zip › Supplementary Tables S1-S3.pdf]

Supplementary Table S1. The composition and nutrient levels of the powder (air dry basis).

Items	Content (g/100 g)
Ingredients	
Swelling soya dregs	51.43
Extruded corn	23.92
Antioxidant	0.12
Beer yeast powder	5.98
KH_2PO_4	0.02
Vitamin premix[1)]	0.36
Sodium citrate	0.60
Choline chloride	0.60
Calcium carbonate	0.30
Irradiated rape pollen	16.67
Total	100
Nutrient levels[2)]	
Crude protein	32.83
Ether extract	6.5
Calcium	0.63
Phosphorus	1.00

1) The premix ingredients (per kilogram premix): VA 1.67 g, VB_1 2.55 g, VB_2 2.36 g, VC 108.67 g, VE 160 g, VD 1.33 g, VB_6 4.42 g, folic acid 6.8 g, nicotinic acid 108.42 g; calcium pantothenate 100 g, phaseomannite 171.82 g, antiseptic 8 g, carrier (corn flour) 323.96 g.

2) The values of crude protein, ether extract, calcium and phosphorus were measured.

Supplementary Table S2. Fatty acid composition of the powder.

Item	Content (mg/100 g)
C16:0	21.4
C18:0	8.96
C18:1n-9c	36.7
C18:2n-6c	19
C18:3n-3	5.21

Supplementary Table S3. Primers for real-time PCR.

Gene	Forward Primer (5'-3')	Reverse Primer (5'-3')
β-actin	TGCCAACACTGTCCTTTCTG	AGAATTGACCCACCAATCCA
rp49	CGTCATATGTTGCCAACTGGT	TTGAGCACGTTCAACAATGG
LOC409515	GTGCTCCTTTGACTCCAGATACTCAG	AGTTGGTCCTCCTACTCTTCCGATAC
LOC409905	CGGTTGTCGTCGGTAGTCCATTG	TTTCTCGTGGTATTCGTCGATCTGTTC
LOC410084	ATCACTGCCACTGCCACCAAAG	GCATAAGCAGCTAAAACAGGAAGACAC
LOC412166	TCAACTCCGCTGCTCATATCTTTGG	TGATAGTTATGCCAACCTTCGCCAAG
LOC413305	AAGAACAGCTATGAAAGCATGGAATGG	GCCAAATAGCAGAAAGTGTCAGAGTTG
LOC551576	AGGCACGCACTTATCTCGAACAAC	TCGAGCATCAGGTACAGAATAAGAACG
LOC552681	TCGGCGTATCAGCGCACTTTATTAC	CGCTGCAATCCTACATGATGTTTCTTG
LOC724933	CAATGGATGGATCTCACTCAAGTAGCC	CACCATAAGCACCTCCTAATCTCCTC
LOC727173	TCTGGAGGAGGCATCACGAGTG	TGGCATAACTGGAGCATTCATGGG
A4	AAGCCAGGCTCAAGCAAACA	GGCTCAGAGATTTCGCAGCTT
FAS	GCAGCCATCCAAGTTGCTCT	AACAGCAGCCATTGCTCCAA
ETF-β	ACACGTGAAGTTGATGGAGGT	CCAGCTTGTCTAACTGGTGGT
ACOX1	CTGCGGAAGATGCCTGGAATCA	TGAAAGCTCGACAATGTGCCTCT
ACOX3	TTCTCGTGTTCGGGCATTAAAGGA	ACCATACCATCCATCACACCCATTT
CPTI	TCATTGCCACGGCTACCATTACC	CGCTGAAGTTTCACACCAATTCCTT
CYP6AS8	ACCGCCAATAAACTCAGAGGAATGT	ACTTCTCGCACATTGACAGGTTCTC
KAT	CTGGCACGTTTGGTGGGATA	TGACCTCCACCAATGCAAGC
